# The chitobiose transporter, *chbC*, is required for chitin utilization in *Borrelia burgdorferi*

**DOI:** 10.1186/1471-2180-10-21

**Published:** 2010-01-26

**Authors:** Ryan G Rhodes, Janet A Atoyan, David R Nelson

**Affiliations:** 1 Department of Cell and Molecular Biology, University of Rhode Island, Kingston, RI 02881, USA; 2 Department of Biological Sciences, University of Wisconsin-Milwaukee, Milwaukee, WI 53211, USA

## Abstract

**Background:**

The bacterium *Borrelia burgdorferi*, the causative agent of Lyme disease, is a limited-genome organism that must obtain many of its biochemical building blocks, including N-acetylglucosamine (GlcNAc), from its tick or vertebrate host. GlcNAc can be imported into the cell as a monomer or dimer (chitobiose), and the annotation for several *B. burgdorferi *genes suggests that this organism may be able to degrade and utilize chitin, a polymer of GlcNAc. We investigated the ability of *B. burgdorferi *to utilize chitin in the absence of free GlcNAc, and we attempted to identify genes involved in the process. We also examined the role of RpoS, one of two alternative sigma factors present in *B. burgdorferi*, in the regulation of chitin utilization.

**Results:**

Using fluorescent chitinase substrates, we demonstrated an inherent chitinase activity in rabbit serum, a component of the *B. burgdorferi *growth medium (BSK-II). After inactivating this activity by boiling, we showed that wild-type cells can utilize chitotriose, chitohexose or coarse chitin flakes in the presence of boiled serum and in the absence of free GlcNAc. Further, we replaced the serum component of BSK-II with a lipid extract and still observed growth on chitin substrates without free GlcNAc. In an attempt to knockout *B. burgdorferi *chitinase activity, we generated mutations in two genes (*bb0002 *and *bb0620*) predicted to encode enzymes that could potentially cleave the β-(1,4)-glycosidic linkages found in chitin. While these mutations had no effect on the ability to utilize chitin, a mutation in the gene encoding the chitobiose transporter (*bbb04*, *chbC*) did block utilization of chitin substrates by *B. burgdorferi*. Finally, we provide evidence that chitin utilization in an *rpoS *mutant is delayed compared to wild-type cells, indicating that RpoS may be involved in the regulation of chitin degradation by this organism.

**Conclusions:**

The data collected in this study demonstrate that *B. burgdorferi *can utilize chitin as a source of GlcNAc in the absence of free GlcNAc, and suggest that chitin is cleaved into dimers before being imported across the cytoplasmic membrane via the chitobiose transporter. In addition, our data suggest that the enzyme(s) involved in chitin degradation are at least partially regulated by the alternative sigma factor RpoS.

## Background

Lyme disease is the most common vector-borne disease in the United States, with almost 250,000 cases reported between 1992 and 2006, and approximately 20,000 new cases reported each year [1]. The disease is contracted from a tick (*Ixodes *species) infected with the spirochete *Borrelia burgdorferi*. *Ixodes *ticks typically feed on small vertebrates such as the white-footed mouse, but humans are sometimes an accidental host. If an infected-feeding tick is not removed before transmission occurs, *B. burgdorferi *disseminates from the site of inoculation and approximately 70% of the time causes a characteristic bulls-eye rash around the site of the tick bite known as erythema migrans. An untreated infection may become systemic and involve connective, neurologic and, to a lesser extent, cardiovascular tissues resulting in clinical complications such as arthritis, encephalitis or atrioventricular block [2]. While antibiotic treatment and tick avoidance are effective in Lyme disease management and prevention, efforts to understand the molecular mechanisms underlying the pathogen's life cycle and host colonization strategies remain important for the development of new prophylactic measures.

*B. burgdorferi *exists exclusively in an enzootic cycle, moving between its tick vector and vertebrate host. In order for the tick to transmit *B. burgdorferi*, it must first obtain the organism from an infected host as spirochetes are not passed transovarially. Once infected, the tick remains so throughout its life-cycle and can pass the bacterium to naïve hosts during subsequent blood meals. Spirochetes exist in low numbers within the unfed-infected tick and are associated with the midgut epithelium, an interaction mediated by outer surface proteins such as OspA and OspB [3-5]. However, as the infected tick takes in a blood meal the number of spirochetes begins to increase. By 24 hours after initiation of the blood meal, bacteria begin to migrate from the tick midgut to the salivary glands where they can be transmitted to a new host [6].

*B. burgdorferi *is a limited-genome organism and relies heavily on its host (tick or vertebrate) for many essential nutrients [7,8]. For example, N-acetylglucosamine (GlcNAc) is required to generate peptidoglycan for cell wall synthesis and may be shuttled into the glycolytic pathway to generate ATP [9]. Spirochetes must obtain GlcNAc from their surrounding environment, and an abundant source of bound GlcNAc is encountered within the tick in the form of chitin. This polymer of alternating GlcNAc residues linked by β-(1,4)-glycosidic bonds functions as a scaffold material for the tick. It is the major component of the exoskeleton and an integral part of the peritrophic membrane [10]. The peritrophic membrane forms as the tick feeds and is composed of chitin, proteins, glycoproteins and proteoglycans. It encases the blood meal and serves as a permeability barrier between the food bolus and the midgut epithelium, enhancing digestion and protecting the midgut epithelium from attack by toxins and pathogens [11-13].

Previous work has demonstrated that *B. burgdorferi *can utilize chitobiose in the absence of free GlcNAc [14-17], and it has been suggested, but not shown, that this bacterium can also utilize longer GlcNAc oligomers (i.e. chitin) [9]. The ability to degrade chitin could potentially serve two purposes for the spirochete within the tick midgut. First, remodeling of the peritrophic membrane during the molt may serve as an important source of GlcNAc in the form of free GlcNAc, chitobiose or longer GlcNAc oligomers [18]. The ability to degrade longer GlcNAc oligomers into chitobiose or free GlcNAc would allow *B. burgdorferi *access to an essential nutrient in the nutrient-poor environment of the unfed tick midgut. Second, studies in *I. ricinus*, the European vector for *B. burgdorferi *sensu lato strains, suggest that the peritrophic membrane in nymphal ticks remains intact for at least 30 days after repletion [19]. Thus, spirochetes apparently must cross the peritrophic membrane in order to successfully colonize the tick midgut epithelium. Studies in *B. burgdorferi *demonstrate that OspA and OspB mediate spirochete association with the tick midgut epithelium shortly after ingestion [3-5], a process that would presumably be facilitated by a chitinase activity. A similar mechanism for vector colonization has been investigated in other organisms that cause vector-borne disease. It has been demonstrated in *Leishmania *[20] and *Plasmodium *[21,22] that chitinases and N-acetylglucosaminidases play a role in weakening the peritrophic membrane, thereby allowing invasion of the midgut epithelium of the sandfly and mosquito, respectively.

Inspection of the *B. burgdorferi *genome reveals both enzymes and transporters that may be involved in chitin degradation. There are two genes predicted to be involved in the cleavage of β-(1,4) glycosidic bonds, a putative β-N-acetylhexosaminidase (*bb0002*) and a putative β-glucosidase (*bb0620*). In addition, previous reports have characterized the chitobiose transport system in *B. burgdorferi*, which is encoded on circular plasmid 26 (*bbb04*, *bbb05 *and *bbb06*) [14,15,17]. It is possible that this transport system plays a role in the utilization of chitin breakdown products (i.e. chitobiose), a mechanism that has been investigated in other chitin-degrading microorganisms [23,24].

As described above, *B. burgdorferi *cannot generate GlcNAc *de novo *and must import this essential sugar from the surrounding environment. Therefore, during *in vitro *propagation the addition of free GlcNAc is necessary for cells to reach optimal cell densities in a single exponential phase. In the absence of free GlcNAc, *B. burgdorferi *exhibits a unique biphasic growth pattern. In the first exponential phase cells utilize the residual GlcNAc and chitobiose present in complex medium components and grow to approximately 2.0 × 10^6 ^cells ml^-1 ^[14,17]. Cells then become starved for GlcNAc and exhibit a death phase in which cell numbers decrease to 1.0 × 10^5 ^cells ml^-1^. By 120 hours cells begin to grow in a second exponential phase and reach cell densities greater than 1.0 × 10^7 ^cells ml^-1^. While the source of GlcNAc in the second exponential phase remains unknown, it is possible that sequestered forms of this sugar such as chitin or glycoproteins present in complex medium components play a role. The goals of this study were to determine if *B. burgdorferi *could utilize chitin as a source of GlcNAc and to identify genes important in the process.

## Results

### Chitinase activity in rabbit serum

Previous reports have described a chitinase activity in mammalian tissues and serum [25-28]. In order to investigate chitin utilization by *B. burgdorferi*, we first determined if there was an inherent chitinase activity in the growth medium (BSK-II) that would interfere with subsequent growth analyses of *B. burgdorferi *in the presence of chitin. To test this, we incubated rabbit serum or BSK-II supplemented with 7% rabbit serum with three artificial fluorescent substrates used to detect chitinase activity: 4-methylumbelliferyl N-acetyl-β-D-glucosaminide (4-MUF GlcNAc), 4-methylumbelliferyl β-D-N,N'-diacetylchitobioside (4-MUF GlcNAc_2_) and 4-methylumbelliferyl β-D-N,N',N"-triacetylchitotrioside (4-MUF GlcNAc_3_). Results demonstrated that rabbit serum has a chitinase activity, as both 4-MUF GlcNAc_2 _and 4-MUF GlcNAc_3 _were cleaved in the presence of serum or with BSK-II supplemented with 7% serum (Table 1). Interestingly, rabbit serum did not cleave the 4-MUF GlcNAc substrate (Table 1), indicating that it does not contain a β-N-acetylglucosaminidase activity. Next, we inactivated the chitinase activity in rabbit serum by boiling so that a chitinase-free medium could be used to evaluate growth of *B. burgdorferi *on chitin substrates. Rabbit serum was diluted (2-fold) with sterile water prior to boiling (see Methods) as undiluted serum solidified when boiled. Boiling for a total of 10 minutes (5 × 2 min) completely inactivated chitinase activity in rabbit serum (Table 1).

**Table 1:** Chitinase activity^a ^in rabbit serum.

Treatment	4-MUF GlcNAc	**4-MUF GlcNAc**_2_	**4-MUF GlcNAc**_3_
	**Average**^b^**(± SE)**^c^	Average(± SE)	Average (± SE)
Serum			
Not Boiled	5.6 (± 3.0)	9,279.7 (± 1,321.6)	17,718.9 (± 6,559.2)
Boiled	5.3 (± 2.2)	12.8 (± 3.6)	16.3 (± 5.2)
BSK + 7% Serum			
Not Boiled	9.3 (± 4.7)	2,610.6 (± 895.5)	2,931.1 (± 170.0)
Boiled	11.0 (± 4.9)	14.3 (± 8.2)	28.2 (± 14.5)

^a ^Chitinase activity was measured as relative fluorescence units

^b ^Average activity of 3 replicate experiments.

^c ^SE, standard error of the mean

### Growth of wild-type *B. burgdorferi *on chitin

**Figure 1:**
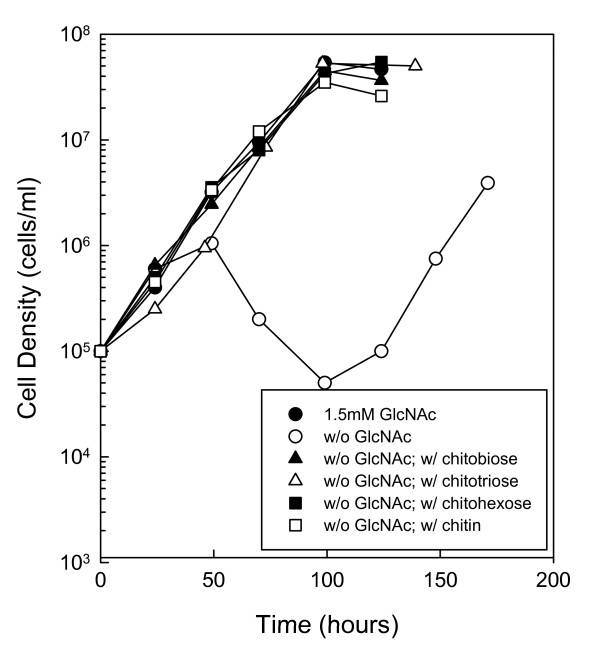
**Chitin utilization in medium supplemented with boiled rabbit serum**. Wild-type cells (B31-A) were cultured in BSK-II without GlcNAc and supplemented with 7% boiled rabbit serum. Late-log phase cells were diluted to 1.0 × 10^5 ^cells ml^-1 ^and the following substrates were added: 1.5 mM GlcNAc (closed circle), No addition (open circle), 75 μM chitobiose (closed triangle), 50 μM chitotriose (open triangle), 25 μM chitohexose (closed square) or 0.4% chitin (open square). Cells were enumerated daily by darkfield microscopy. This is a representative experiment that was repeated five times.

Inactivating the chitinase activity in rabbit serum allowed us to perform growth experiments to determine if *B. burgdorferi *possesses a chitinase activity and can utilize chitin in the absence of free GlcNAc. Previous reports by our laboratory [17] and others [14,15] demonstrated that *B. burgdorferi *exhibits biphasic growth when cultured in the absence of free GlcNAc, and that chitobiose can substitute for free GlcNAc resulting in growth to maximum cell density in a single exponential phase. We repeated those experiments here using BSK-II lacking GlcNAc and supplemented with 7% boiled rabbit serum. As shown in Fig. 1, boiling the serum did not have an adverse effect on cell growth. In addition, when cells were cultured in the presence of 50 μM chitotriose, 25 μM chitohexose or 0.4% coarse chitin flakes, maximum cell densities were reached in a single exponential phase, similar to growth on 1.5 mM GlcNAc or 75 μM chitobiose (Fig. 1). These results demonstrate for the first time that *B. burgdorferi *can use GlcNAc oligomers (longer than chitobiose) and chitin in the absence of free GlcNAc.

**Figure 2:**
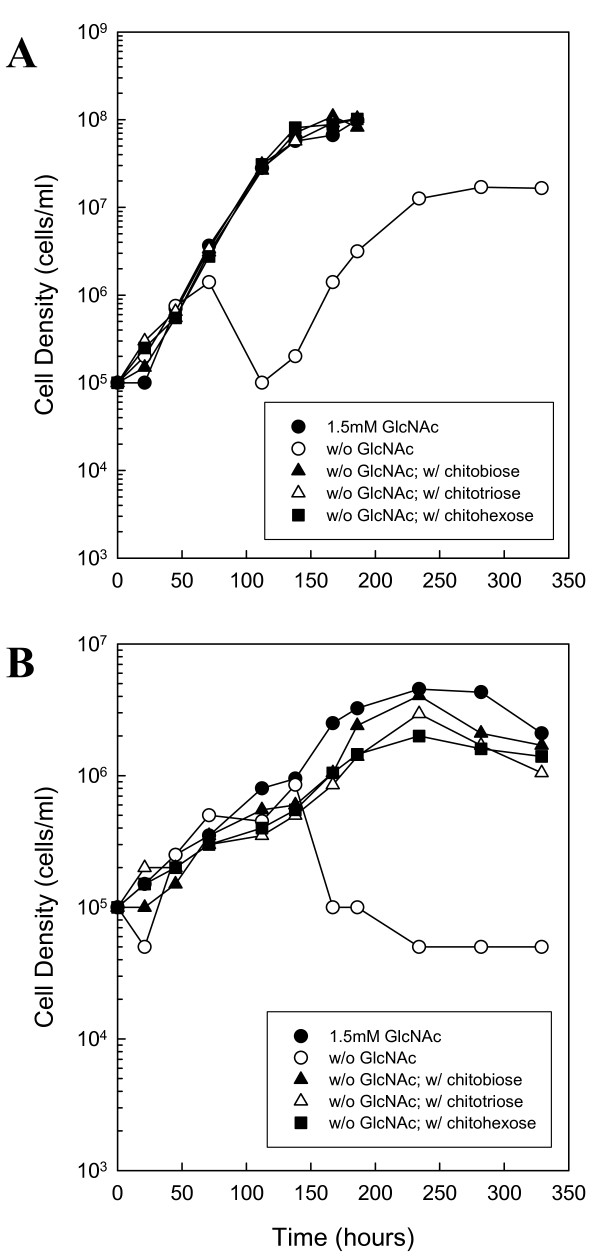
**Chitin utilization in boiled medium without BSA**. BSK-II without GlcNAc and BSA was supplemented with 7% rabbit serum. Wild-type cells were cultured in unboiled medium (A) or medium that was boiled for 10 min (B). Late-log phase cells were diluted to 1.0 × 10^5 ^cells ml^-1 ^and the following substrates were added: 1.5 mM GlcNAc (closed circle), No addition (open circle), 75 μM chitobiose (closed triangle), 50 μM chitotriose (open triangle) or 25 μM chitohexose (closed square). Cells were enumerated daily by darkfield microscopy. This is a representative experiment that was repeated three times.

**Figure 3:**
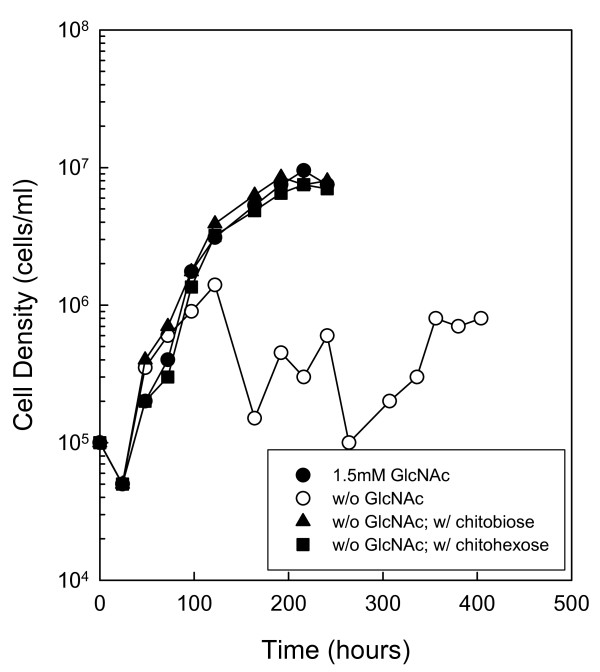
**Chitin utilization in serum-free medium containing a lipid supplement**. Serum-free BSK-II was supplemented with a lipid mixture. Wild-type cells in late-log phase were diluted to 1.0 × 10^5 ^cells ml^-1 ^in the absence of free GlcNAc and supplemented with the following substrates: 1.5 mM GlcNAc (closed circle), No addition (open circle), 75 μM chitobiose (closed triangle) or 25 μM chitohexose (closed square). Cells were enumerated daily by darkfield microscopy. This is a representative experiment that was repeated three times.

We conducted two additional growth experiments in which either the entire medium was inactivated by boiling (Fig. 2) or the serum was removed altogether (Fig. 3). First, BSK-II was prepared without bovine serum albumin (BSA) and supplemented with 7% rabbit serum (not boiled). Removing the BSA from the medium allowed us to boil BSK-II with 7% rabbit serum without the medium solidifying. The medium was boiled (5 × 2 min) to inactivate serum chitinase activity, and the growth experiment described above was repeated. Removing the BSA from the medium did not noticeably change cell growth (compare Fig. 2A with Fig. 1). In contrast, boiling the medium did slow cell growth with maximum cell densities decreased by more than one order of magnitude (Fig. 2B). However, cells still showed the same growth pattern for chitin utilization as described above, suggesting that they could use chitotriose and chitohexose in the absence of free GlcNAc.

In another set of growth experiments, rabbit serum was replaced with a lipid supplement previously described by Cluss *et al *[29] to rule out the possibility of residual chitinase activity in boiled serum that was not detected by the artificial fluorescent substrates. Cells were subcultured at least twice in a medium containing the lipid supplement prior to initiating growth experiments without GlcNAc. Growth of wild-type cells in serum-free BSK-II lacking GlcNAc and supplemented with 1.5 mM GlcNAc, 75 μM chitobiose or 25 μM chitohexose resulted in a single exponential phase and a maximum cell density of approximately 1.0 × 10^7 ^cells ml^-1 ^(Fig. 3). While the maximum cell density was approximately one order of magnitude lower than in BSK-II containing 7% boiled rabbit serum, the growth pattern was the same as that observed previously with chitin substrates (compare Fig. 3 with Fig. 1). Of note, cells cultured without GlcNAc in this serum-free medium only reached a maximum cell density of 8.0 × 10^5 ^cells ml^-1 ^in the second exponential phase, which is more than one order of magnitude lower than that observed in medium containing 7% serum.

### Growth of a β-N-acetylhexosaminidase and β-glucosidase double mutant on chitin

**Figure 4:**
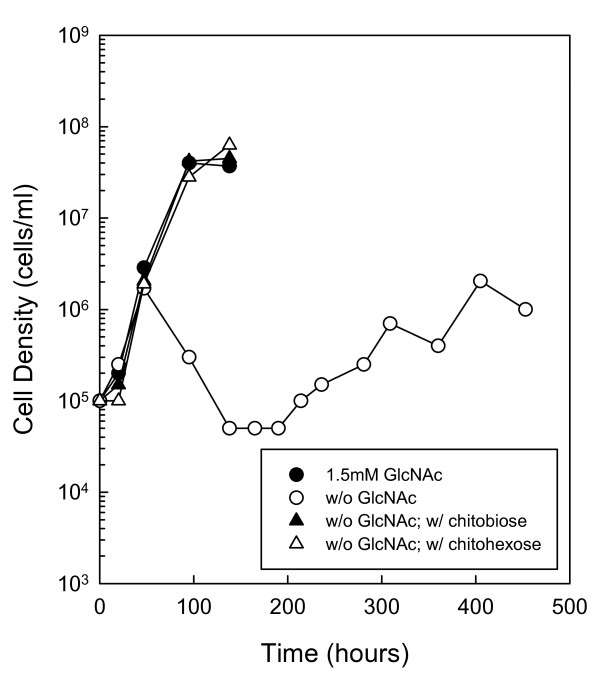
**β-N-acetylhexosaminidase (*bb0002*) and β-glucosidase (*bb0620*) double mutant utilizes chitin**. Growth of RR60 (double mutant) in the presence of chitobiose or chitohexose. Late-log phase cells were diluted to 1.0 × 10^5 ^cells ml^-1 ^in BSK-II containing 7% boiled serum, lacking GlcNAc and supplemented with the following substrates: 1.5 mM GlcNAc (closed circle), No addition (open circle), 75 μM chitobiose (closed triangle) or 25 μM chitohexose (open triangle). Cells were enumerated daily by darkfield microscopy. This is a representative experiment that was repeated twice.

*bb0002 *(putative β-N-acetylhexosaminidase) and *bb0620 *(putative β-glucosidase) are the only obvious genes annotated in the *B. burgdorferi *genome that encode enzymes potentially involved in the degradation of chitin. We generated mutations in *bb0002 *and *bb0620 *to determine if eliminating the function of either or both of these genes would result in a defect in chitobiose or chitin utilization (see Methods). Both of the single mutant strains and the double mutant strain were cultured in BSK-II containing 7% boiled rabbit serum, lacking GlcNAc and supplemented with 75 μM chitobiose or 25 μM chitohexose. As expected from a previous report [14], the *bb0002 *mutant (RR04) showed no defect in chitobiose utilization, and no defect in the ability of this mutant to utilize chitohexose was observed (data not shown). Similar results were also obtained for the *bb0620 *mutant, RR53 (data not shown). The double mutant (RR60) also showed no defect in chitobiose or chitohexose utilization (Fig. 4), suggesting that either these genes are not involved in chitin degradation or that a redundant activity is encoded elsewhere in the genome. We also attempted to generate mutants in two genes with LysM motifs (*bb0262 *and *bb0761*) since LysM domains are involved in binding to peptidoglycan and chitin, typically through the GlcNAc moiety [30]. We constructed a *bb0761 *mutant, but it showed no defect in utilization of GlcNAc oligomers when cultured in BSK-II lacking GlcNAc and supplemented with 7% boiled rabbit serum and chitobiose or chitohexose (data not shown). Several attempts to generate a *bb0262 *mutant were unsuccessful suggesting this may be an essential gene due to a role in cell wall synthesis or remodeling.

### Growth of a *chbC *mutant on chitin

**Figure 5:**
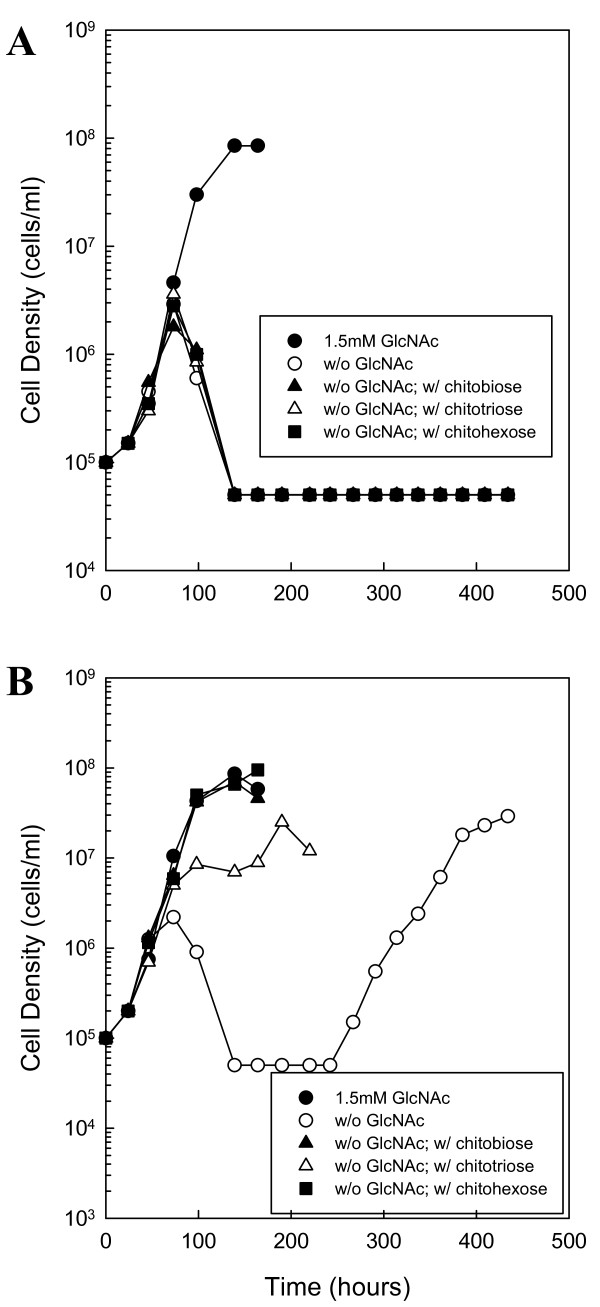
**Growth of a *chbC *mutant and complemented mutant on chitin**. (A) Growth of RR34 (*chbC *mutant) in the presence of chitobiose, chitotriose and chitohexose. Late-log phase cells were diluted to 1.0 × 10^5 ^cells ml^-1 ^in BSK-II containing 7% boiled serum, lacking GlcNAc and supplemented with the following substrates: 1.5 mM GlcNAc (closed circle), No addition (open circle), 75 μM chitobiose (closed triangle), 50 μM chitotriose (open triangle) or 25 μM chitohexose (closed square). Cells were enumerated daily by darkfield microscopy. (B) Growth of JR14 (RR34 complemented with BBB04/pCE320) in the presence of chitobiose, chitotriose and chitohexose. Late-log phase cells were diluted to 1.0 × 10^5 ^cells ml^-1 ^in BSK-II containing 7% boiled serum, lacking GlcNAc and supplemented with the following substrates: 1.5 mM GlcNAc (closed circle), No addition (open circle), 75 μM chitobiose (closed triangle), 50 μM chitotriose (open triangle) or 25 μM chitohexose (closed square). Cells were enumerated daily by darkfield microscopy. These are representative growth experiments that were repeated four times.

Previous work demonstrated that *B. burgdorferi *uses a phosphotransferase system (PTS) to import chitobiose, and *bbb04 *(*chbC*) encodes the transporter for this system [14,15]. We wanted to determine if *chbC *is necessary for chitin utilization in *B. burgdorferi*, as chitobiose transport has been shown to be important in the chitin utilization pathways of other organisms [24,31]. To test this, a *chbC *deletion mutant was generated (RR34) and cultured in BSK-II containing 7% boiled rabbit serum without GlcNAc and supplemented with either 75 μM chitobiose, 50 μM chitotriose or 25 μM chitohexose (Fig. 5A). Under all conditions RR34 failed to grow to optimal cell densities, and only reached 1.8 - 3.6 × 10^6 ^cells ml^-1 ^before blebbing and entering a death phase. In contrast, wild-type cells with a functional *chbC *transporter grew to maximal cell densities without exhibiting a death phase, when cultured without free GlcNAc and supplemented with chitotriose or chitohexose (compare Fig. 5A with Figs. 1 and 2). In addition, RR34 did not exhibit a second exponential phase when cultured in the absence of free GlcNAc for 434 hours, whether or not GlcNAc oligomers were present. These results strongly suggest that *chbC*, and by extension chitobiose transport, is necessary for chitin utilization by *B. burgdorferi*.

To confirm that *chbC *is necessary for growth on chitin and second exponential phase growth in the absence of free GlcNAc, we created a complementation plasmid to restore wild-type function. The complemented *chbC *mutant (JR14) was cultured in BSK-II containing 7% boiled rabbit serum, lacking free GlcNAc and supplemented with 75 μM chitobiose, 50 μM chitotriose or 25 μM chitohexose (Fig. 5B). Comparison of the wild type (Fig. 1), the *chbC *mutant (Fig. 5A), and the *chbC*-complemented mutant (Fig. 5B) under these growth conditions demonstrate that the presence of the functional *chbC *gene in the complemented mutant restored wild-type growth on all three substrates in the absence of free GlcNAc.

### Growth of an *rpoS *mutant on chitin

**Figure 6:**
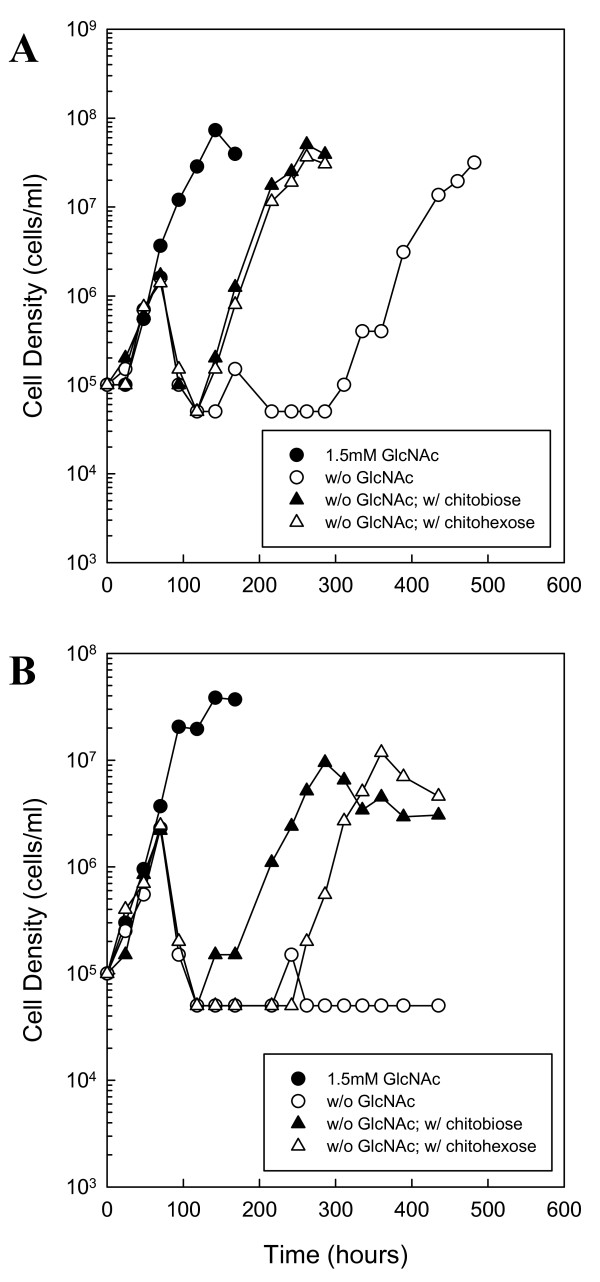
**RpoS regulates chitobiose and chitin utilization**. Growth of A74 (*rpoS *mutant) in BSK-II without GlcNAc and supplemented with 7% unboiled (A) or boiled serum (B). Late-log phase cells were diluted to 1.0 × 10^5 ^cells ml^-1 ^and cultures were supplemented with the following substrates: 1.5 mM GlcNAc (closed circle), No addition (open circle), 75 μM chitobiose (closed triangle) or 25 μM chitohexose (open triangle). Cells were enumerated daily by darkfield microscopy. This is a representative experiment that was repeated three times.

Previous work in our laboratory demonstrated that the alternative sigma factor RpoS partially regulates chitobiose utilization, by regulating the expression of *chbC *during GlcNAc starvation [17]. Since *chbC *is necessary for chitin utilization, we hypothesized that RpoS may also be involved in the regulation of other genes in this pathway. To test this, we cultured an *rpoS *mutant (A74) in BSK-II without free GlcNAc, supplemented with 75 μM chitobiose or 25 μM chitohexose and containing either 7% unboiled (Fig. 6A) or boiled (Fig. 6B) rabbit serum. As in our previous report [17], culturing the *rpoS *mutant with chitobiose in the absence of free GlcNAc resulted in biphasic growth. This was observed in the presence of both unboiled (Fig. 6A) and boiled (Fig. 6B) rabbit serum with the second exponential phase starting at 142 hours in either medium. Comparison of chitohexose utilization by the *rpoS *mutant in unboiled (Fig. 6A) or boiled (Fig. 6B) serum revealed biphasic growth under both conditions, but with a delay in the initiation of the second exponential growth phase only in a medium supplemented with boiled serum. The delay in second exponential phase growth ranged from 72 to 120 h in the three replicate experiments conducted. These data suggest a role for RpoS in the regulation of chitin utilization separate from its role in regulating *chbC *expression.

## Discussion

Chitin is one of the most abundant polymers in the environment [32] and is a major structural component of arthropods, including Ixodid ticks, the vector hosts for *B. burgdorferi*. *B. burgdorferi *must obtain GlcNAc from its tick and vertebrate hosts and does so by transporting either free GlcNAc or chitobiose into the cell [14-17]. Recently, Tilly *et al *[14,15] reported that *B. burgdorferi *cells exhibit biphasic growth in the absence of free GlcNAc *in vitro*. It was proposed that the second growth phase observed during GlcNAc starvation was due to the up regulation of *chbC *and the utilization of chito-oligomers present in the yeastolate component of BSK-II [14]. While we were able to confirm that the induction of *chbC *expression during GlcNAc starvation is responsible for chitobiose utilization, our observations suggested that yeastolate is not the source of sequestered GlcNAc for second exponential phase growth [17]. Thus, we set out to determine if *B. burgdorferi *could utilize chitin given that it is a major component of the tick peritrophic membrane [11-13]. Chitin utilization could prove beneficial to spirochetes in the nutrient-limited environment of the unfed-infected tick midgut and aid in the colonization of the midgut epithelium.

Prior to conducting growth studies in the presence of chitin, we determined if there was an inherent chitinase activity present in the medium. Previous reports characterized chitinase activity in goat serum [25], guinea pig blood [26], human macrophages [27] and a variety of mouse tissues [28]. While chitinase activity has not been previously described in rabbit serum, the evolutionary conservation of this enzymatic activity in rodents and primates [33] suggested that it may also be present in rabbit serum. We demonstrated heat-sensitive chitinase activity in rabbit serum (Table 1). In addition, rabbit serum showed no activity against 4-MUF GlcNAc, suggesting that it possesses chitinase activity but not a β-N-acetylglucosaminidase activity in which free GlcNAc is released from the non-reducing end of chitin. These results support our observation that the source of sequestered GlcNAc in the second exponential phase is not due to chito-oligomers present in the yeastolate component of BSK-II [17]. Any chito-oligomers present in yeastolate would be degraded to chitobiose by the chitinase activity present in rabbit serum, and imported into the cells by the *chbC *transporter.

To determine whether *B. burgdorferi *could utilize chitin and GlcNAc oligomers longer than chitobiose, we either inactivated the chitinase activity in rabbit serum by boiling before adding it to BSK-II or we replaced the rabbit serum with a lipid extract. In both cases, *B. burgdorferi *cells provided with chitin or various chitin oligomers as the sole source of GlcNAc grew in one exponential phase to optimal cell densities (Figs. 1 and 3). In the absence of these added sources of GlcNAc, the cells failed to grow to high cell densities. These data strongly suggest that *B. burgdorferi *has the genes necessary to degrade and utilize chitin or GlcNAc oligomers in the absence of free GlcNAc.

Additionally, GlcNAc starvation in the absence of rabbit serum resulted in biphasic growth, but with a lower maximum cell density in the second exponential phase (Fig. 3). This suggests that rabbit serum and one or more other components in BSK-II contribute the sequestered GlcNAc necessary for growth in the second exponential phase, possibly in the form of glycoproteins or glycosaminoglycans. It is interesting to note that boiling the serum or the entire medium had an impact on the ability of cells to grow in a second exponential phase in some experiments (Fig. 2B and Fig. 4). For example, in boiled medium without BSA, cells did not exhibit a second exponential phase in the absence of free GlcNAc (Fig. 2B). In another case, a reduced growth rate and a reduced cell density in the second exponential phase was observed for RR60 (the *bb0002 *and *bb0620 *double mutant) in the absence of free GlcNAc (Fig. 4). However, results with RR60 do not lead us to conclude that either of these genes play a significant role in obtaining sequestered GlcNAc in the second exponential phase, because the wild-type strain grew to the same final cell density as RR60 in this experiment (data not shown). Additionally, RR60 was cultured in BSK-II lacking GlcNAc and supplemented with serum that was not boiled, and cells grew to > 1.0 × 10^7 ^cells ml^-1 ^in the second exponential phase (data not shown). The lack of a second exponential phase observed in boiled BSK-II (Fig. 2B) and the slower second exponential phase accompanied by reduced cell density observed with RR60 (Fig. 4) was occasionally observed and seemed to correlate with different batches of boiled medium or serum. This suggests that prolonged boiling alters components within the serum that *B. burgdorferi *normally utilizes for second exponential phase growth.

In addition to growth experiments, we attempted to detect *B. burgdorferi *chitinase activity using the artificial fluorescent substrates described above (data not shown). We used both culture supernatants and cell lysates from cultures starved for GlcNAc and supplemented with 7% boiled rabbit serum and various GlcNAc oligomers or chitin. While cells grew to maximum cell densities as expected, we were unable to detect cleavage of any of the artificial fluorescent substrates. These results were surprising in light of the growth experiments (Figs. 1, 2 and 3) and the known ability of *B. burgdorferi *to utilize chitobiose [14-17]. It is possible that the enzyme activity expressed was below the detection limit of our assay or that the artificial substrates were not recognized by these enzymes.

While attempts to knockout chitinase activity in this study were not successful, we did identify other candidates by genome analysis. We examined genes annotated by The Institute for Genomic Research (TIGR; http://cmr.jcvi.org) as hypothetical or conserved hypothetical using the NCBI Conserved Domain Database (CDD; http://www.ncbi.nlm.nih.gov/sites/entrez?db=cdd) to target those genes with domains that could be involved in chitin degradation or chitin binding. We generated a list of potential targets that included five genes with a potential hydrolase domain (*bb0068*, *bb0168*, *bb0421*, *bb0504 *and *bb0511*), three with a potential Lysin Motif (LysM; *bb0262*, *bb0323 *and *bb0761*), one with a potential Goose Egg White Lysozyme domain (GEWL; *bb0259*) and one with a cyclodextrin transglycosylase domain (CGTase; *bb0600*). As noted above, the *bb0761 *mutant showed no defect in utilization of GlcNAc oligomers and attempts to generate a *bb0262 *mutant were unsuccessful suggesting this is an essential gene with a role in cell wall synthesis or remodeling.

A recent report on *Ralstonia *A-471 described a novel goose egg white-type lysozyme gene with chitinolytic activity [34]. BLAST analysis of the catalytic domain against the *B. burgdorferi *genome shows 36% identity at the amino acid level (E-value is 7.9e^-08^) to *bb0259*, which has a GEWL domain. We did not attempt to knockout this gene, but it may be a target to consider in future studies.

Since chitobiose transport is important for chitin utilization in other organisms [24,31], we evaluated the role of *chbC *during chitin utilization in *B. burgdorferi*. As expected from previous studies [14,17], RR34 (*chbC *mutant) was unable to grow on chitobiose in the absence of free GlcNAc (Fig. 5A). Similarly, no growth was observed when RR34 cells were cultured in the absence of GlcNAc and supplemented with chitotriose or chitohexose, demonstrating that *chbC *is also required for the utilization of GlcNAc oligomers longer than chitobiose. Complementation of the *chbC *mutant by introduction of the wild-type *chbC *gene on a shuttle vector (Fig. 5B) restores the wild-type phenotype. Together, these results demonstrate that chitobiose transport is necessary for the utilization of chitobiose and longer GlcNAc oligomers, and suggest that an unidentified enzyme(s) involved in the degradation of chitin is secreted, either extracellularly or into the periplasm. In addition, these results show that chitobiose transport is necessary for utilization of sequestered GlcNAc in the second exponential phase, and support our hypothesis that GlcNAc oligomers are not the source of sequestered GlcNAc in the second exponential phase.

Previous work conducted in our laboratory suggested that RpoS, one of two alternative sigma factors present in *B. burgdorferi*, regulates chitobiose utilization in the B31-A background by partially regulating expression of *chbC *during GlcNAc starvation [17]. Here we cultured an *rpoS *mutant in BSK-II lacking GlcNAc and supplemented with chitobiose or chitohexose and 7% unboiled (Fig. 6A) or boiled (Fig. 6B) rabbit serum. Biphasic growth of the *rpoS *mutant in the presence of chitobiose was nearly identical in unboiled and boiled rabbit serum. This is important because it further demonstrates that unboiled serum does not possess a β-N-acetylglucosaminidase activity that cleaves chitobiose to monomeric GlcNAc. In contrast, growth of the *rpoS *mutant supplemented with chitohexose was delayed in boiled serum compared to that in unboiled rabbit serum. This delay supports the data presented in Table 1 showing an inherent chitinase activity in unboiled rabbit serum as *rpoS *mutant growth on chitohexose in unboiled serum (Fig. 6A) mirrors that on chitobiose, suggesting the chitinase activity in the rabbit serum degraded the chitohexose to chitobiose. In addition, the delay in chitohexose utilization in boiled serum strongly suggests that RpoS regulates chitin utilization not only through the regulation of *chbC *[17], but also through the regulation of other gene(s) important for degradation of chitin. Recently, Caimano *et al *[35] characterized the RpoS regulon in the 297 c162 background after temperature-shift *in vitro *and after maintenance in dialysis membrane chambers in rats or rabbits. We were unable to find any of our candidate chitin utilization genes upon examination of differentially regulated genes identified in their study. It is possible that starvation for GlcNAc is necessary for the induction of these genes, a condition that was not tested by Caimano *et al*.

In this study we provide evidence that *B. burgdorferi *can utilize GlcNAc oligomers and chitin in the absence of free GlcNAc, and we show that chitobiose transport via *chbC *is required for utilization of these substrates. A previous report suggested *chbC *is not required for maintenance or transmission of the organism between ticks and mice [15]. However, these studies were conducted in a controlled laboratory environment using pathogen-free ticks and mice. It is possible *chbC *plays a role in infection in a natural setting by providing a competitive advantage to spirochetes in colonizing ticks that are often colonized with more than one microorganism. In addition, *chbC *is required for obtaining sequestered GlcNAc during second exponential phase growth *in vitro *which most likely comes from glycoproteins or glycosaminoglycans, so there may also be a role for this transporter in the mammal. However, it is also possible that chitinase activity, rather than chitin utilization, is required for transmission, as chitinase activity may be important for penetration of the peritrophic membrane and colonization of the tick midgut. In this instance, the *chbC *gene may be retained, but chitobiose uptake and utilization may be of secondary importance.

## Conclusions

In this study we provide evidence of an inherent chitinase activity in rabbit serum, a component of the *B. burgdorferi *growth medium, BSK-II. We inactivated this activity by boiling, and showed that cells can utilize GlcNAc oligomers and chitin as a source of GlcNAc in the presence of boiled serum or a lipid supplement. In addition, we demonstrated that transport of chitobiose via the chitobiose transporter, *chbC*, is required for chitin utilization by this organism. Finally, delayed growth of an *rpoS *mutant on chitohexose suggests that this alternative sigma factor is involved in the regulation of chitin utilization.

## Methods

### Bacterial strains and culture conditions

Bacterial strains and plasmids described in this work are listed in Table 2. *B. burgdorferi *strains were maintained in modified BSK-II [36] supplemented with 7% rabbit serum and any necessary antibiotics (see Table 2). BSK-II was modified by replacing 10× CMRL-1066 with 10× Media 199 (Invitrogen Corp.; Carlsbad, CA). Some experiments were conducted with boiled rabbit serum to inactivate the inherent chitinase activity. Serum was diluted 2-fold in sterile deionized water, incubated in a boiling water bath for 2 min and allowed to cool to room temperature. Incubation in the boiling water bath was repeated for a total of 5 times and 14% of boiled serum was added to BSK-II (final serum concentration was 7%). In certain growth experiments serum was replaced with a lipid supplement stock of 26 μM cholesterol, 12 μM palmitic acid and 12 μM oleic acid [29]. Lipids were transferred to BSK-II as an ethanolic mixture at a final concentration of 0.1% (vol/vol). Plasmids were maintained in *E. coli *DH5α that was cultured in lysogeny broth (LB; 1% tryptone, 0.5% yeast extract, 1% NaCl) containing the appropriate antibiotic(s) (see Table 2). Antibiotics were used at the following concentrations for *B. burgdorferi *strains: streptomycin, 100 μg ml^-1^; coumermycin A1, 0.5 μg ml^-1^; kanamycin, 340 μg ml^-1^. Antibiotics were used at the following concentrations for *E. coli *DH5α: streptomycin 100 μg ml^-1^; kanamycin, 50 μg ml^-1^; ampicillin, 200 μg ml^-1^.

**Table 2:** Strains and plasmids used in this study.

Strain or Plasmid	Genotype and Description	Reference
Strains		
*B. burgdorferi*		
B31-A	High passage non-infectious wild type	[42]
RR04	Str^R^; B31-A putative β-N-acetylhexosaminidase (*bb0002*) mutant	This study
RR53	Kan^R^; B31-A putative β-glucosidase (*bb0620*) mutant	This study
RR60	Str^R ^Kan^R^; B31-A double mutant for *bb0002 *and *bb0620*	This study
RR34	Str^R^; B31-A *chbC *mutant	This study
JR14	Str^R ^Kan^R^; RR34 complemented with BBB04/pCE320	This study
A74	Coum^R^; B31-A *rpoS *mutant	[42]
*E. coli*		
DH5α	*supE*44 F^- ^Δ*lac*U169 (ϕ80*lac*Z ΔM15) *hsdR*17 *relA*1 *endA*1*gyrA*96 *thi*-1*relA*1	[43]
Plasmids		
pKFSS1	Str^R^; *B. burgdorferi *shuttle vector, cp9 based	[37]
pBSV2	Kan^R^; *B. burgdorferi *shuttle vector, cp9 based	[38]
pCE320	Kan^R ^Zeo^R^; *B. burgdorferi *shuttle vector, cp32 based	[40]
pBB0002.7	Str^R^; *aadA*::*bb0002*	This study
pBB0620.5	Kan^R^; *kan*::*bb0620*	This study
pBBB04.5	Str^R^; *aadA*::*bbb04*	This study
BBB04/pCE320	Kan^R^; *bbb04 *complementation construct	This study

### Generation of a β-N-acetylglucosaminidase (bb0002) and β-glucosidase (bb0620) double mutant in *B. burgdorferi*

To generate a *bb0002*/*bb0620 *double mutant of *B. burgdorferi *we first generated single mutations for each gene by deletion of 63 and 81 bp, respectively, and insertion of an antibiotic resistance gene (streptomycin or kanamycin) as a selectable marker. The construct used to generate the *bb0002 *mutant with streptomycin resistance was created as follows: (i) a 1.2 kb fragment of the 3' end of *bb0002 *and flanking sequence was amplified from B31-A genomic DNA using primers with engineered restriction sites, 5'BB0002mutF (KpnI) and 5'BB0002mutR (XbaI) (for a list of primers used in this study see Table 3); (ii) the amplicon was TA cloned into pCR2.1 (Invitrogen, Corp.) to generate pBB0002.3; (iii) pBB0002.3 and pKFSS1 [37] (a *B. burgdorferi *shuttle vector conferring streptomycin resistance; Table 2) were digested with KpnI and XbaI and separated by gel electrophoresis; (iv) the 1.2 kb fragment from pBB0002.3 was gel extracted using the QIAquick PCR Purification Kit (Qiagen, Inc.; Valencia, CA) according to the manufacturer's instructions, and cloned into the gel extracted fragment from pKFSS1 to create pBB0002.4; (v) the 1.2 kb fragment and flanking streptomycin resistance cassette from pBB0002.4 was PCR amplified using TaKaRa ExTaq (Fisher Scientific; Pittsburgh, PA) and the primers 5'BB0002mutF (KpnI) and pKFSS1 R1; (vi) the resulting 2.7 kb amplicon was TA cloned into pGEM T-Easy (Promega, Inc.; Madison, WI) to generate pBB0002.5A or B (based on orientation of the PCR product insertion); (vii) a pBB0002.5B clone in which the 3' end of the streptomycin resistance cassette was adjacent to the XmaI site in the pGEM T-Easy vector was identified by restriction digest; (viii) the 5' end of *bb0002 *and flanking DNA was amplified using primers 3'BB0002mutF (XmaI) and 3'BB0002mutR (SacII), and TA cloned into pCR2.1 to create pBB0002.6; (ix) pBB0002.5B and pBB0002.6 were digested with XmaI and SacII and separated by gel electrophoresis; (x) the 2.0 kb fragment from pBB0002.6 was gel extracted, and cloned into the gel extracted fragment from pBB0002.5B to create the final construct, pBB0002.7. In summary, 63 bp of the *bb0002 *gene was deleted and the streptomycin cassette under control of the *B. burgdorferi *P_*flgB *_promoter (from pKFSS1) was inserted in the opposite orientation.

**Table 3:** Oligonucleotide primers used in this study

Primer Name	Sequence (5'→3')
5'BB0002mutF (KpnI)	GCTAGGGTACCACATTGCCTTTATCGGAATATTGACATC
5'BB0002mutR (XbaI)	GCTAGTCTAGAAAGATGCGGAGCAGACAAAGGGAT
pKFSS1 R1	TGATGAACAGGGTCACGTCGTC
3' BB0002mutF (XmaI)	GCTAGCCCGGGCGATATTAAGCTCTTGAACATTCTTAAA
3'BB0002mutR (SacII)	GCTAGCCGCGGTAGTGCTATTAGTGCTTTATCTTTATTG
5'BB0620mutF3 (KpnI)	GCTAGGGTACCTACTTTGAATTTTGAATATGGAG
5'BB0620mutR2 (SalI)	GCTAGGTCGACTACCCAAATCAATCAATCAC
pBSV2 R1	TTATTATCGTGCACTCCTCCCGGT
3'BB0620mutF2 (SacII)	GCTAGCCGCGGCGTATCCCAAAAATCAATAGAAAA
3'BB0620mutR2 (AatII)	GCTAGGACGTCATGCAATCACCGCAATAGAAGCGG
5'BBB04mutF2 (BamHI)	GCTAGGGATCCGAATAAGTAGCTTTACGTCT
5'BBB04mutR2 (PstI)	GCTAGCTGCAGTACCAACAGTGGTATGTTGA
3'BBB04mutF1 (XmaI)	GCTAGCCCGGGCCAATTTTGCTAGCAATAGGA
3'BBB04mutR1 (SacII)	GCTAGCCGCGGGCATCTGGATTTAGGTCTGCTTTGA
BBB04 complement F1	GCTTCATTACTTCAACAGGACGACG
BBB04 complement R1	TCGCTAAGGCGTGTCTCAGCAATA
*chbC *F1	GGGAATTCAGCCCAATTCATGGTTTCC
*chbC *R1	GGCGGAACAGACTCTGGAAGCTTAAT
BB0002 CF1	ATGGACTTTTTAAAAACCTTTTCTTTTTTGTTTTTTAGC
BB0002 CR1	CTAAGGAATGAGTACTATATTGACACCCGA
BB0620 mut confirm F1	TCAAGAGTGGTATTGCCGTGTCCT
BB0620 mut confirm R1	ACTTGAACCCACGACAACTCGGAT
BBB04 mut confirm F1	AGCAGCATCTCCACCGTAAGGTAT
BBB04 mut confirm R1	CACCAGAGTAAGCTACAACAGGCA

The construct used to generate the *bb0620 *mutant with kanamycin resistance was created as follows: (i) a 2.7 kb fragment of the 3' end of *bb0620 *and flanking sequence was amplified using primers 5'BB0620mutF3 (KpnI) and 5'BB0620mutR2 (SalI); (ii) the amplicon was TA cloned into pCR2.1 to generate pBB0620.1; (iii) pBB0620.1 and pBSV2 [38] (a *B. burgdorferi *shuttle vector conferring kanamycin resistance; Table 2) were digested with KpnI and SalI and separated by gel electrophoresis; (iv) the 2.7 kb fragment from pBB0620.1 was gel extracted and cloned into the gel extracted fragment from pBSV2 to create pBB0620.2; (v) the 2.7 kb fragment and flanking kanamycin resistance cassette was PCR amplified using primers 5'BB0620mutF3 and pBSV2 R1; (vi) the resulting 4.3 kb amplicon was TA cloned into pGEM T-Easy to create pBB0620.3A or B (based on orientation of the PCR product insertion); (vii) a pBB0620.3B clone was identified by restriction digest in which the 3' end of the kanamycin resistance cassette was adjacent to the SacII restriction site in the pGEM T-Easy vector; (viii) the 5' end of *bb0620 *and flanking DNA was amplified using primers 3'BB0620mutF2 (SacII) and 3'BB0620mutR2 (AatII) and TA cloned into pCR2.1 to create pBB0620.4; (ix) pBB0620.3B and pBB0620.4 were digested with SacII and AatII and separated by gel electrophoresis; (x) the 1.7 kb fragment from pBB0620.4 was gel extracted and cloned into the gel extracted fragment from pBB0620.3B to create the final construct, pBB0620.5. In summary, 81 bp near the 5' end of *bb0620 *were deleted and the kanamycin cassette under control of the *B. burgdorferi *P_*flgB *_promoter (from pBSV2) was inserted in the opposite orientation.

All plasmid constructs described above were confirmed by restriction digestion and/or sequence analysis. Plasmids pBB0002.7 and pBB0620.5 were used to generate deletion/insertion mutations in B31-A. Specifically, plasmids were concentrated to greater than 1 μg μl^-1 ^and 10 μg of each plasmid was introduced into separate competent B31-A preparations by electroporation. Cells from each transformation reaction were resuspended in 10 ml of BSK-II containing 20 μg ml^-1 ^phosphomycin, 50 μg ml^-1 ^rifampicin and 2.5 μg ml^-1 ^amphotericin B (Antibiotic Mixture for *Borrelia *100×; Sigma-Aldrich; St. Louis, MO), and allowed to recover overnight (18-24 h) prior to plating. Cells were plated on BSK-II containing either 100 μg ml^-1 ^streptomycin (pBB0002.7) or 340 μg ml^-1 ^kanamycin (pBB0620.5) according to the protocol of Samuels *et al *[39]. Antibiotic resistant colonies appearing 10-14 d after plating were transferred to liquid BSK-II and cell lysates were screened by PCR using primers flanking the antibiotic insertion site. One clone for each mutation was chosen for growth experiments. The *bb0002 *mutant was designated RR04, and the *bb0620 *mutant was designated RR53. Mutations in RR04 and RR53 were confirmed by PCR amplification of genomic DNA using primers flanking the antibiotic insertion site [Additional file 1 and Additional file 2], and DNA sequencing confirmed insertion of the antibiotic resistance gene.

To generate the *bb0002*/*bb0620 *double mutant, competent RR04 cells were transformed with 10 μg of pBB0620.5. Cells were resuspended in BSK-II and allowed to recover overnight prior to plating on BSK-II containing 100 μg ml^-1 ^streptomycin and 340 μg ml^-1 ^kanamycin. PCR was used to screen the transformants and a clone containing mutations in both genes was designated RR60. In addition, PCR was conducted on genomic DNA obtained from this clone using primers flanking the antibiotic insertion site [Additional file 1 and Additional file 2], DNA sequencing of the PCR products confirmed insertion of the antibiotic resistance genes in *bb0002 *and *bb0620*.

### Construction of a *chbC *mutant in *B. burgdorferi*

The construct used to generate a *chbC *(*bbb04*) deletion/insertion in B31-A was created as follows: (i) a 2.6 kb fragment of the 3' end of *chbC *and flanking DNA was amplified using primers 5'BBB04mutF2 (BamHI) and 5'BBB04mutR2 (PstI); (ii) the amplicon was TA cloned into pCR2.1 to generate pBBB04.1; (iii) pBBB04.1 and pKFSS1 were digested with BamHI and PstI and separated by gel electrophoresis; (iv) the 2.6 kb fragment from pBBB04.1 was gel extracted and cloned into the gel extracted fragment from pKFSS1 to generate pBBB04.2; (v) the 2.6 kb fragment and flanking streptomycin resistance cassette in pBBB04.2 were PCR amplified using primers 5'BBB04mutF2 (BamHI) and pKFSS1 R1; (vi) the resulting 4.0 kb amplicon was TA cloned into pGEM T-Easy to generate pBBB04.3A or B (based on orientation of the PCR product insertion); (vii) a pBBB04.3B was identified by restriction digest in which the 3' end of the streptomycin resistance cassette was adjacent to the XmaI site in the pGEM T-Easy vector; (viii) the 5' end of *bbb04 *and flanking DNA was amplified using primers 3'BBB04mutF1 (XmaI) and 3'BBB04mutR1 (SacII) and TA cloned into pCR2.1 to create pBBB04.4; (ix) pBBB04.3B and pBBB04.4 were digested with XmaI and SacII and separated by gel electrophoresis; (x) the 1.8 kb fragment from pBBB04.4 was gel extracted and cloned into the gel extracted fragment from pBBB04.3B to create the final construct, pBBB04.5. In summary, 141 bp near the 5' end of *chbC *were deleted and the streptomycin resistance gene under the control of the *B. burgdorferi *P_flgB_promoter (from pKFSS1) was inserted in the opposite orientation. All plasmid constructs were confirmed by restriction digestion and DNA sequencing.

The *chbC *deletion/insertion mutation was generated by transforming B31-A with 10 μg of pBBB04.5 and plating on BSK-II containing 100 μg ml^-1 ^streptomycin as described above. Transformants were selected with streptomycin and screened by PCR using primers flanking the antibiotic insertion site. A single clone, RR34, was chosen for subsequent growth experiments and the mutation was confirmed by PCR with primers flanking the antibiotic insertion site [Additional file 3]. DNA sequencing was performed on the PCR product confirming the insertion of the streptomycin resistance gene.

### Complementation of the *chbC *mutant

To complement the *chbC *mutant (RR34) the wild-type *chbC *gene (*bbb04*) and flanking DNA was amplified from B31-A genomic DNA using primers BBB04 complement F1 and BBB04 complement R1. The resulting 3.0 kb fragment was TA cloned into pCR2.1 to generate pchbCcomp.1. Next, pchbCcomp.1 and pBSV2 [38] were digested with SacI and XbaI and separated by gel electrophoresis. The 3.0 kb fragment from pchbCcomp.1 was gel extracted and cloned into the gel extracted fragment from pBSV2 to create the complementation construct pchbCcomp.2. Several attempts were made to complement RR34 with pchbCcomp.2; however, no clones were obtained. Therefore, we transferred the *bbb04 *fragment from pchbCcomp.2 to pCE320 [40], a *B. burgdorferi *shuttle vector with a circular plasmid 32 (cp32) origin of replication, by digesting with NotI. The new construct, designated BBB04/pCE320, was transformed into RR34 and plated on BSK-II containing 100 μg ml^-1 ^streptomycin and 340 μg ml^-1 ^kanamycin as described above. One clone, designated JR14, was selected for further experiments, and PCR confirmation showed this clone carried both mutant and wild-type copies of *chbC *[Additional file 3].

### Nucleotide sequencing and computer analysis

Nucleic acid sequencing was performed by the University of Rhode Island Genomics and Sequencing Center using a 3130xl Genetic Analyzer (Applied Biosystems; Forest City, CA). Sequencing reactions were prepared using the BigDye^® ^Terminator v3.0 Cycle Sequencing Kit. Sequences were analyzed using the DNASTAR Lasergene software (DNASTAR, Inc.; Madison, WI).

### Chitinase activity assay

Chitinase activity assays were performed as previously described [41] using the following substrates: 4-MUF GlcNAc, 4-MUF GlcNAc_2 _and 4-MUF GlcNAc_3 _(Sigma-Aldrich). Briefly, 200 μl reactions were prepared by combining 150 μl Tris buffered saline (TBS; 25 mM Tris, 150 mM NaCl), 30 μl of sample and 20 μl of the appropriate substrate (1 mM stock solution in DMSO) in a black 96 well microtiter plate with a clear bottom (Fisher Scientific). Plates were incubated at 33°C for up to 48 h, and fluorescence was monitored using the SpectraMax2 fluorimeter (Molecular Devices Corp.; Sunnyvale, CA) with excitation at 390 nm and emission at 485 nm.

### Growth curves

For growth experiments, late-log phase cells (5.0 × 10^7 ^to 1.0 × 10^8 ^cells ml^-1^) cultured in complete BSK-II were diluted to 1.0 × 10^5 ^cells ml^-1 ^in 6 ml of BSK-II lacking GlcNAc. Typically, 6-12 μl of culture was transferred to 6 ml of fresh medium; therefore, negligible amounts of nutrients were transferred with the inoculum. Cultures were supplemented with 1.5 mM GlcNAc, 75 μM chitobiose, 50 μM chitotriose, 25 μM chitohexose (V-Labs; Covington, LA) or 0.04% (w/v) chitin flakes from crab shells (Sigma-Aldrich). Chitin oligomers were > 95% pure as determined by the manufacturer. For experiments in which BSK-II was supplemented with boiled serum or lipid extract, cells were subcultured (i.e. diluted 1:1000) in fresh medium containing the appropriate supplement at least two times prior to the initiation of growth experiments. Therefore, the initial inoculum from BSK-II containing serum that was not boiled was diluted 10^9^- fold in BSK-II supplemented with boiled serum or lipid extract before the initiation of growth experiments. All growth experiments were carried out at 33°C and 3% CO_2_. To enumerate cells, 2.5 μl of culture was transferred to a Petroff-Hauser counting chamber (Hauser Scientific; Horsham, PA) and cells were counted in all 25 squares by darkfield microscopy. For cultures with a cell density greater than 1.0 × 10^7 ^cells ml^-1 ^a 10-fold dilution in BSK-II was made prior to loading in the counting chamber. Each growth curve is representative of multiple independent trials, as data could not be pooled due to the length of experiments and the different times at which bacteria were enumerated.

## Abbreviations

GlcNAc: N-acetylglucosamine; BSK-II: Barbour-Stoenner-Kelly medium; 4-MUF GlcNAc: 4-methylumbelliferyl N-acetyl-β-D-glucosaminide; 4-MUF GlcNAc_2_: 4-methylumbelliferyl β-D-N,N'-diacetylchitobioside; 4-MUF GlcNAc_3_: 4-methylumbelliferyl β-D-N,N',N"-triacetylchitotrioside; BSA: bovine serum albumin; PTS: phosphotransferase system; LB: lysogeny broth; Coum^R^: coumermycin A_1 _resistant; Kan^R^: kanamycin resistant; Ery^R^: erythromycin resistant; Str^R^: streptomycin resistant.

## Authors' contributions

RGR and DRN conceived of the study. RGR performed the fluorescent chitinase assays, growth curve analyses, generated the RR mutants listed in Table 2 and drafted the manuscript. JAA constructed JR14 and performed growth curve analyses. DRN supervised the work and edited the manuscript. All authors read and approved the final manuscript.

## Supplementary Material

Additional file 1 **PCR Confirmation of putative β-N-acetylhexosaminidase (*bb0002*) mutants**. PCR confirmation of the *bb0002 *deletion/insertion mutation in RR04 (*bb0002 *mutant) and RR60 (*bb0002 *and *bb0620 *double mutant). Click here for file

Additional file 2 **PCR Confirmation of β-glucosidase mutations**. PCR confirmation of the *bb0620 *deletion/insertion mutation in RR53 (*bb0620 *mutant) and RR60 (*bb0002 *and *bb0620 *double mutant). Click here for file

Additional file 3 **PCR confirmation of *chbC *(*bbb04*) mutation and complementation**. PCR confirmation of RR34 (*bbb04 *deletion/insertion mutant) and JR14 (RR34 complemented with pBBB04/pCE320). Click here for file
