# Supplementary material for: The chitobiose transporter, chbC, is required for chitin utilization in Borrelia burgdorferi
Source: BMC Microbiol. 2010 Jan 26;10:21. doi: 10.1186/1471-2180-10-21 (PMC2845121; doi:10.1186/1471-2180-10-21)
Supplement: Additional file 1 — PCR Confirmation of putative β-N-acetylhexosaminidase (bb0002) mutants. PCR confirmation of the bb0002 deletion/insertion mutation in RR04 (bb0002 mutant) and RR60 (bb0002 and bb0620 double mutant). [file 1471-2180-10-21-S1.DOC]

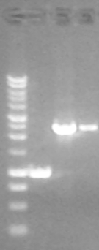


1

2

3

4

0.25

1.0

10.0

3.0

2.0

Kbp

**Additional File 1. PCR Confirmation of β-N-acetylhexosaminidase (*bb0002*) mutations.**

PCR confirmation of the *bb0002* deletion/insertion mutation in RR04 (*bb0002* mutant) and RR60 (*bb0002* and *bb0620* double mutant) using primers BB0002 CF1 and BB0002 CR1 which flank the insertion site. The larger PCR product is 2419 bp (upper arrow) and contains the streptomycin resistance gene within *bb0002*, and the smaller PCR product representing the wild-type gene is 1029 bp (lower arrow): lane 1 – 1-kb ladder, lane 2 – B31-A genomic DNA, lane 3 – RR04 genomic DNA, lane 4 – RR60 genomic DNA.
