# Supplementary material for: The chitobiose transporter, chbC, is required for chitin utilization in Borrelia burgdorferi
Source: BMC Microbiol. 2010 Jan 26;10:21. doi: 10.1186/1471-2180-10-21 (PMC2845121; doi:10.1186/1471-2180-10-21)
Supplement: Additional file 2 — PCR Confirmation of β-glucosidase mutations. PCR confirmation of the bb0620 deletion/insertion mutation in RR53 (bb0620 mutant) and RR60 (bb0002 and bb0620 double mutant). [file 1471-2180-10-21-S2.DOC]

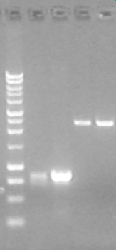


1

2

3

4

5

0.25

1.0

10.0

3.0

2.0

Kbp

**Additional File 2. PCR Confirmation of β-glucosidase mutations.**

PCR confirmation of the *bb0620* deletion/insertion mutation in RR53 (*bb0620* mutant) and RR60 (*bb0002* and *bb0620* double mutant) using primers BB0620 mut confirm F1 and BB0620 mut confirm R1 which flank the insertion site. The larger PCR product is 2463 bp (upper arrow) and contains the kanamycin resistance gene within *bb0620*, and the smaller PCR product representing the wild-type gene is 862 bp (lower arrow): lane 1 – 1-kb ladder, lane 2 – B31-A genomic DNA, lane 3 – RR04 genomic DNA (*bb0002* single mutant), lane 4 – RR53 genomic DNA, lane 5 – RR60 genomic DNA.
