# Supplementary material for: The chitobiose transporter, chbC, is required for chitin utilization in Borrelia burgdorferi
Source: BMC Microbiol. 2010 Jan 26;10:21. doi: 10.1186/1471-2180-10-21 (PMC2845121; doi:10.1186/1471-2180-10-21)
Supplement: Additional file 3 — PCR confirmation of chbC (bbb04) mutation and complementation. PCR confirmation of RR34 (bbb04 deletion/insertion mutant) and JR14 (RR34 complemented with pBBB04/pCE320). [file 1471-2180-10-21-S3.DOC]

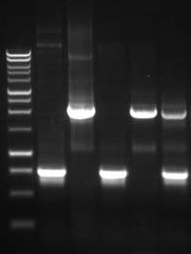


0.25

1.0

2.0

3.0

10.0

Kbp

1

2

3

4

5

6

**Additional File 3. PCR confirmation of *chbC* (*bbb04*) mutation and complementation.**

PCR confirmation of RR34 (*bbb04* deletion/insertion mutant) and JR14 (RR34 complemented with pBBB04/pCE320) using primers BBB04 mut confirm F1 and BBB04 mut confirm R1 which flank the insertion site. The larger PCR product is 2078 bp (upper arrow) and contains the streptomycin resistance gene within *bbb04*. The smaller PCR product is 760 bp (lower arrow) and represents the wild-type *bbb04* gene: lane 1 – 1-kb ladder, lane 2 – B31-A genomic DNA, lane 3 – pBBB04.5 (mutation construct), lane 4 – BBB04/pCE320 (complementation plasmid), lane 5 – RR34 genomic DNA (*chbC* mutant), lane 6 – JR14 genomic DNA (RR34 complemented with BBB04/pCE320).
